# Supplementary material for: Dynamic Structure of Yeast Septin by Fast Fluctuation-Enhanced Structured Illumination Microscopy
Source: Microorganisms. 2021 Oct 29;9(11):2255. doi: 10.3390/microorganisms9112255 (PMC8620077; doi:10.3390/microorganisms9112255)
Supplement: Supplementary file 1 [file microorganisms-09-02255-s001.zip › si/Supplementary information-20210924 .docx]

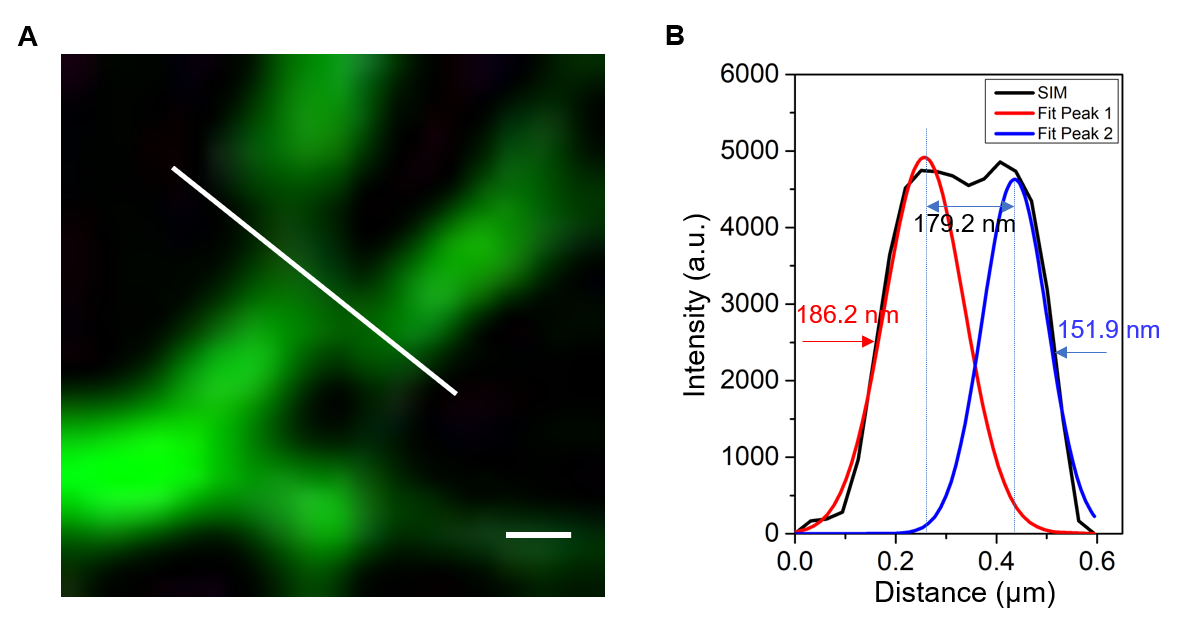


**Figure S1. Confirmation of SIM resolution under the same conditions.** Related to Figure 1.(A) Enlarged images of the white-boxed region in figure 1(B). (B) Intensity profiles along the white lines in (A). The double-sided arrows indicate the peak-to-peak distance. Red and blue lines indicate the Gaussian fit used to determine the FWHM (one-way arrows) (Scale bar, 100 nm).


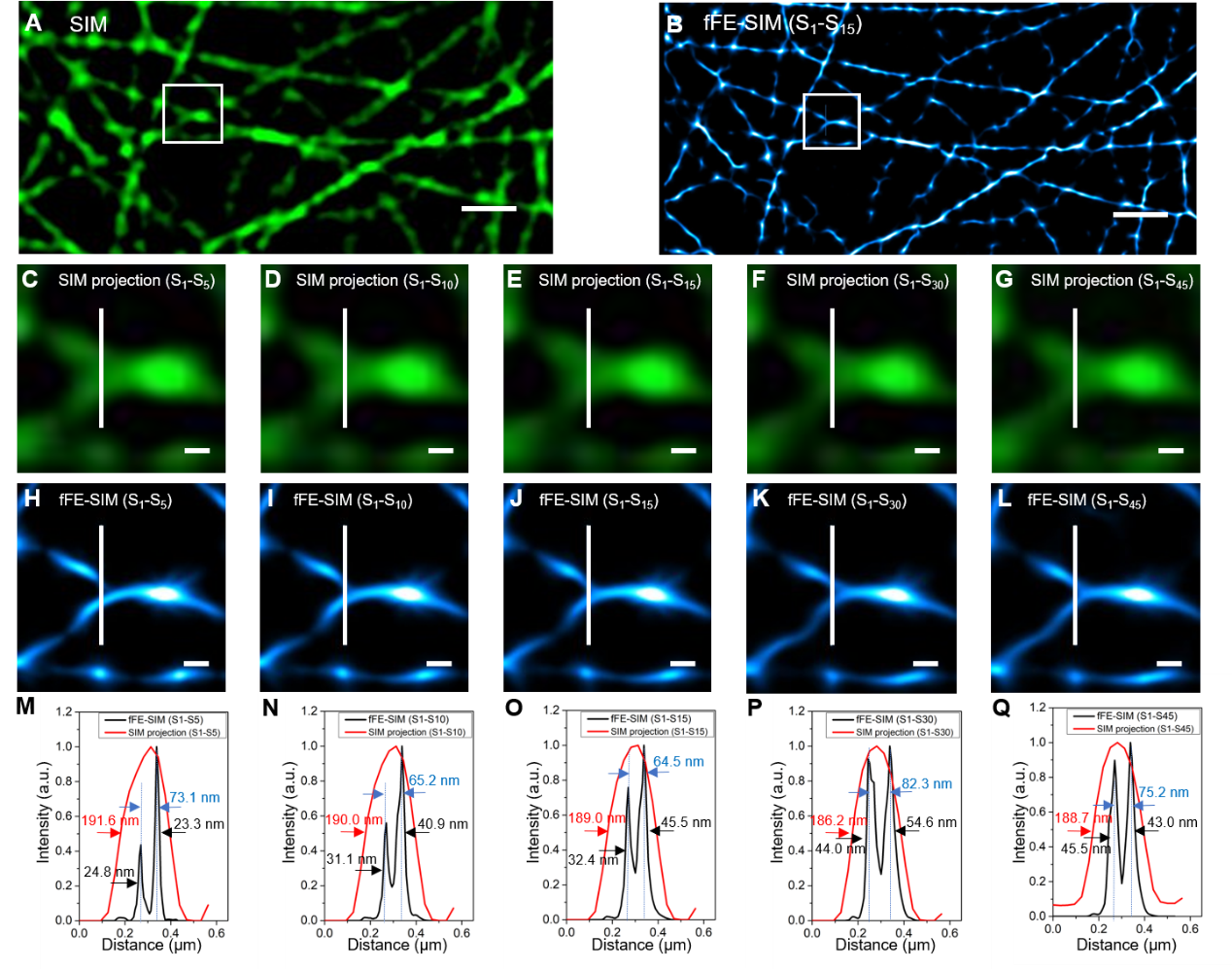


**Figure S2. Another example of Figure 1.** Related to Figure 1.(A) Image of subcellular microtubules labeled with EGFP by SIM algorithm processing (scale bar, 1 µm). (B) Reconstructed fFE-SIM image by 15 frames of SIM images (scale bar, 1 µm). (C-G) Sum projections by 5,10,15,30 and 45 SIM images indicated by the white box in (b) respectively (scale bar, 200 nm). (H-I) Reconstructed fFE-SIM image by 5,10,15,30 and 45 SIM images indicated by the white box in (A) respectively (scale bar, 200 nm). (M-Q) Corresponding line-scanning profile from the SIM projection images (red) and fFE-SIM images (black) processing by 5,10,15,30 and 45 SIM images respectively. The red and black one-way arrows indicate FWHM. The blue arrows indicate the peak-to-peak distance.


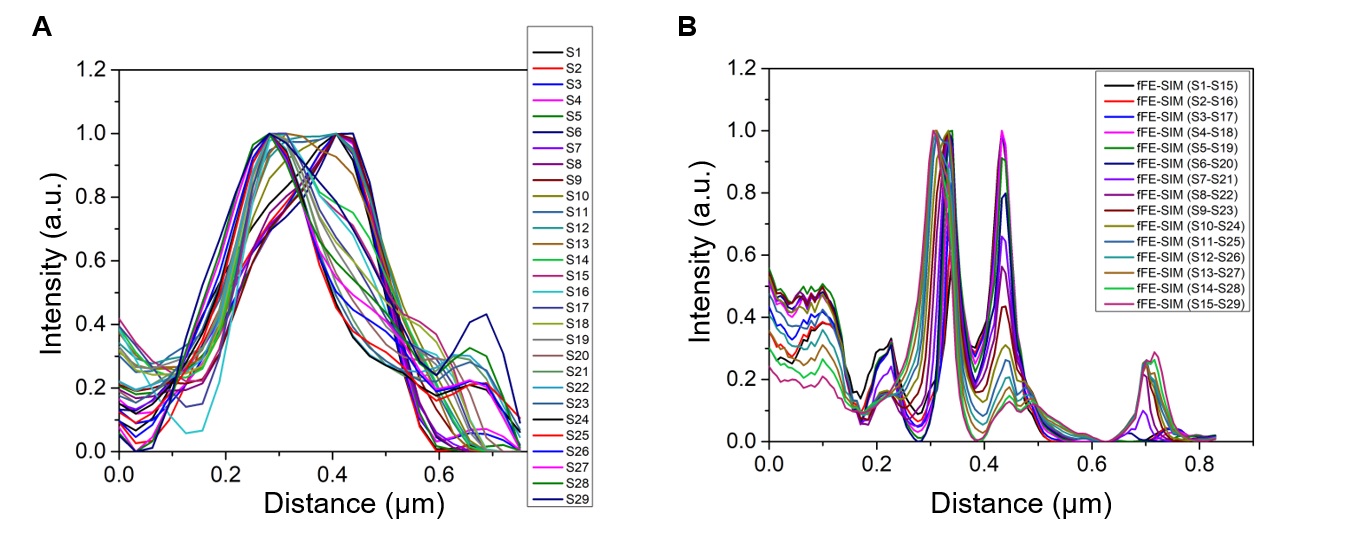


**Figure S3. All data in Figure 2 (C, G).** Related to Figure 2.


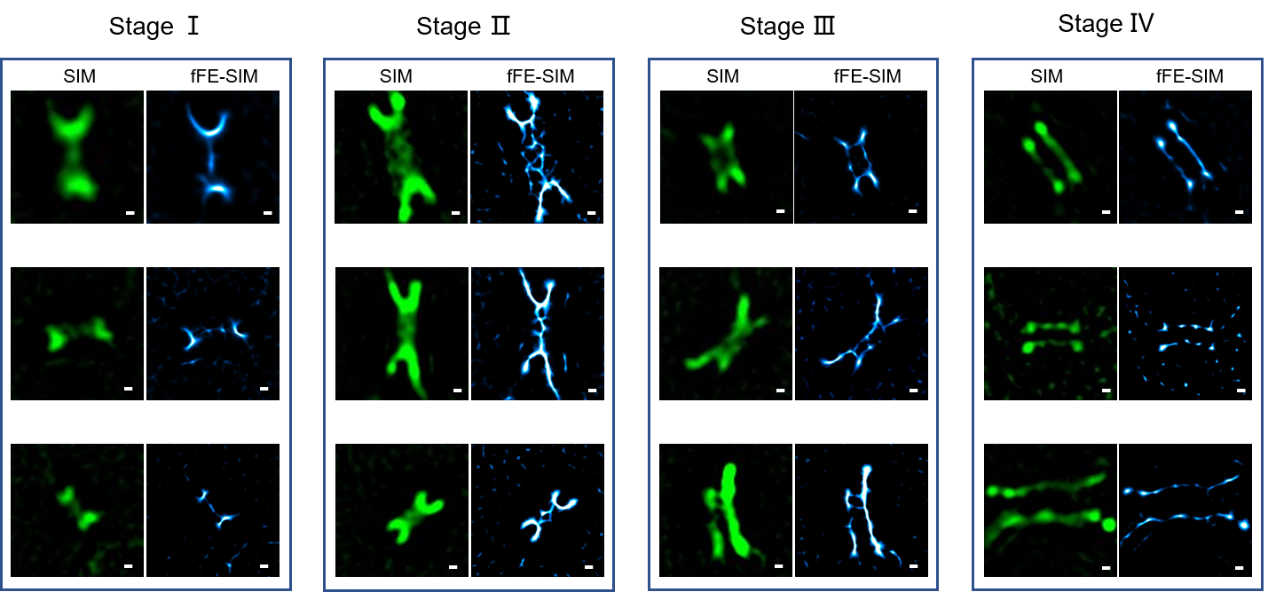


**Figure S4. The hourglass to double ring transition visualized by SIM and fFE-SIM.** Related to Figure 4.Three more examples of GFP-Cdc12 at each stage (scale bar, 200 nm).
