# Supplementary figures and images for: Dynamic Structure of Yeast Septin by Fast Fluctuation-Enhanced Structured Illumination Microscopy
Source: Microorganisms. 2021 Oct 29;9(11):2255. doi: 10.3390/microorganisms9112255 (PMC8620077; doi:10.3390/microorganisms9112255)

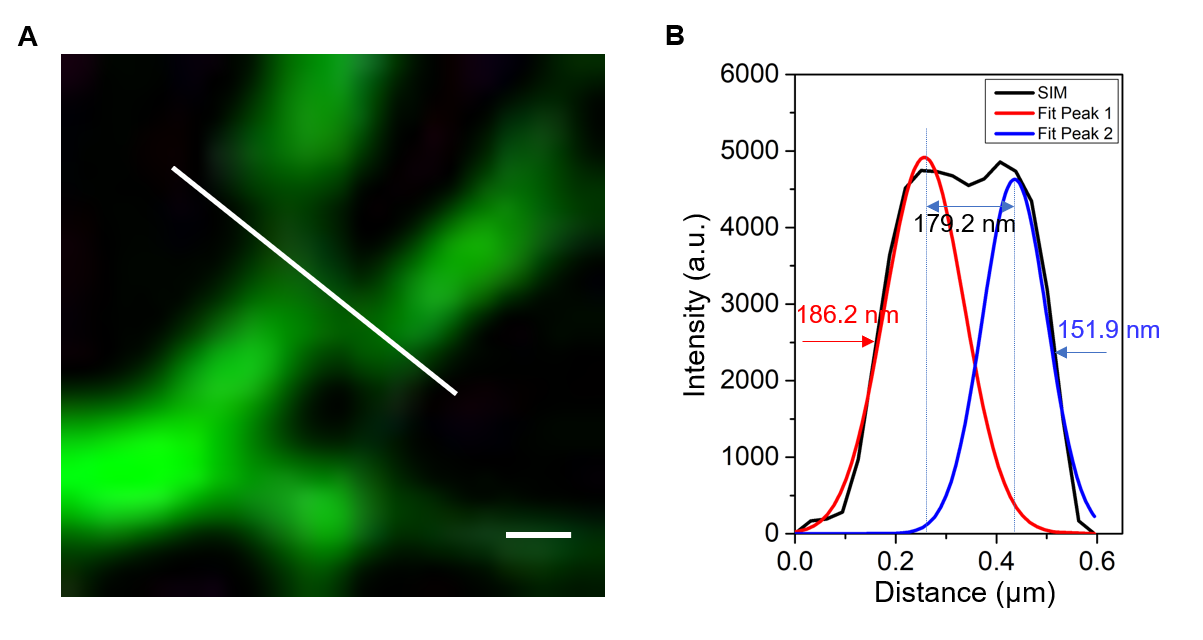

Supplement: Supplementary file 1 [file microorganisms-09-02255-s001.zip › si/S1.tif]

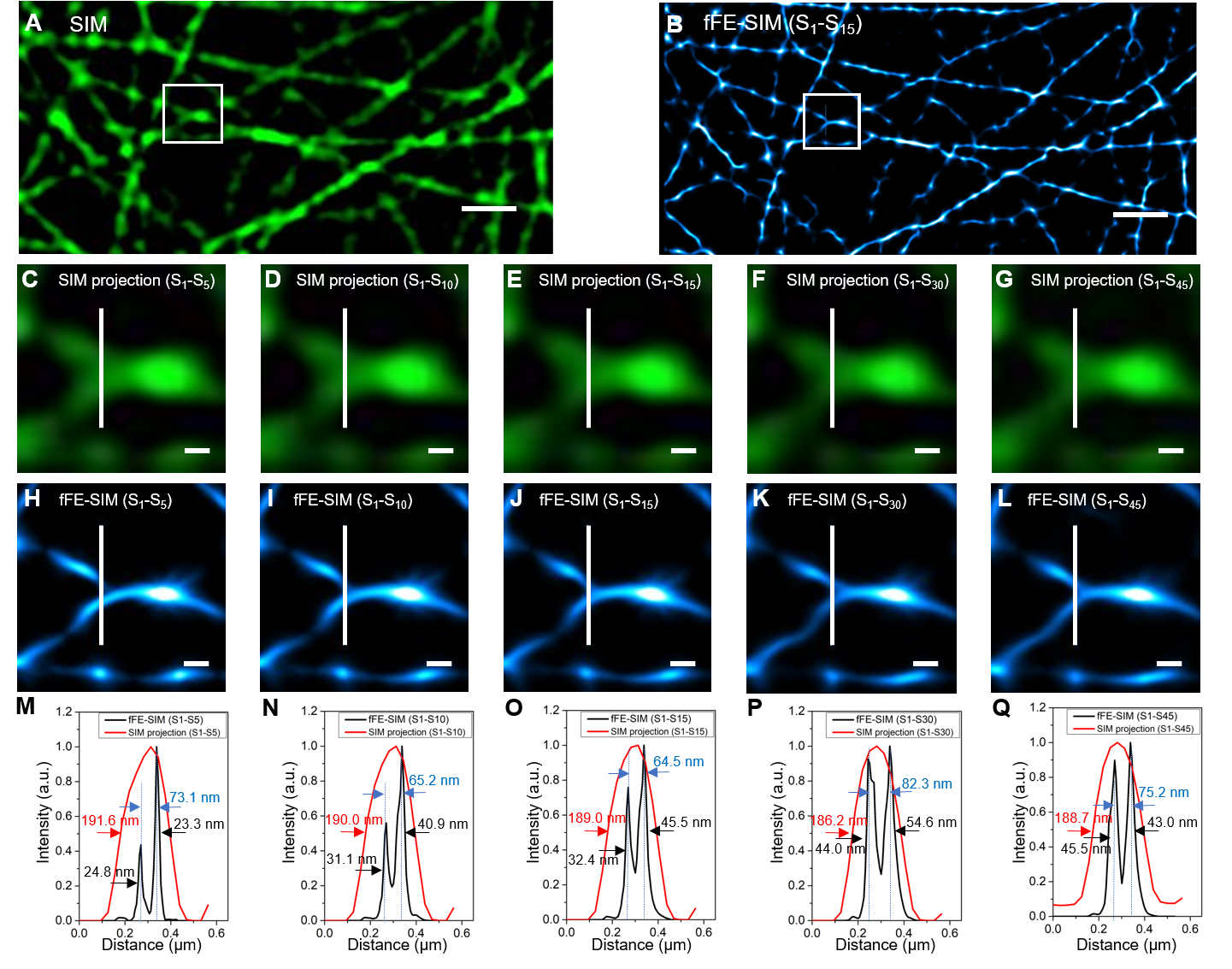

Supplement: Supplementary file 1 [file microorganisms-09-02255-s001.zip › si/S2.tif]

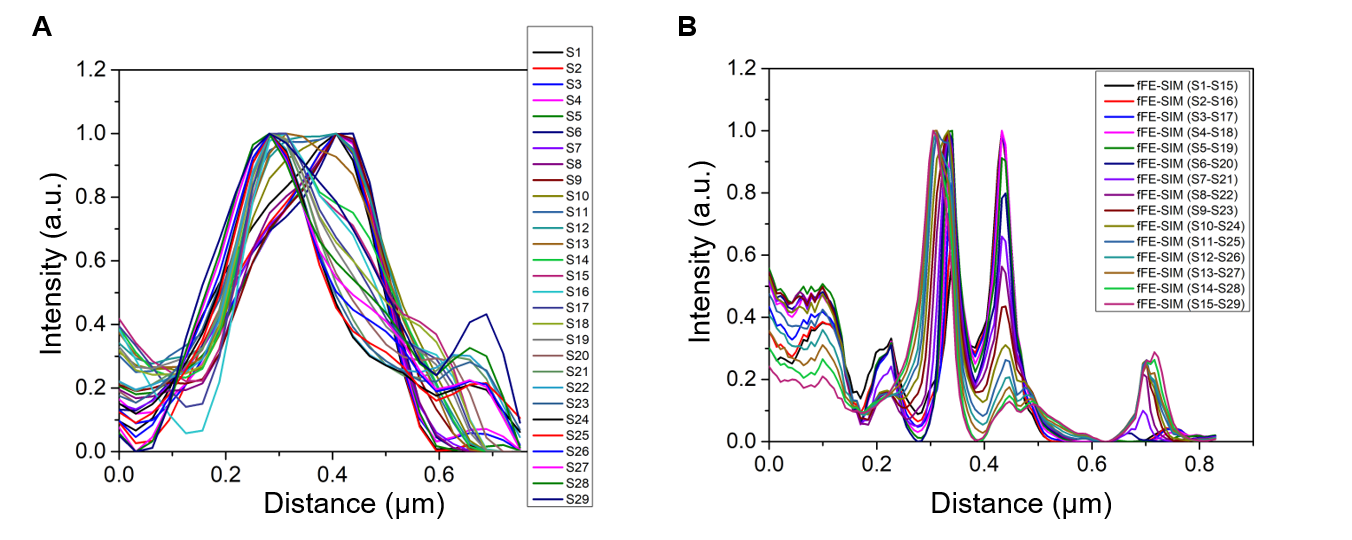

Supplement: Supplementary file 1 [file microorganisms-09-02255-s001.zip › si/S3.tif]

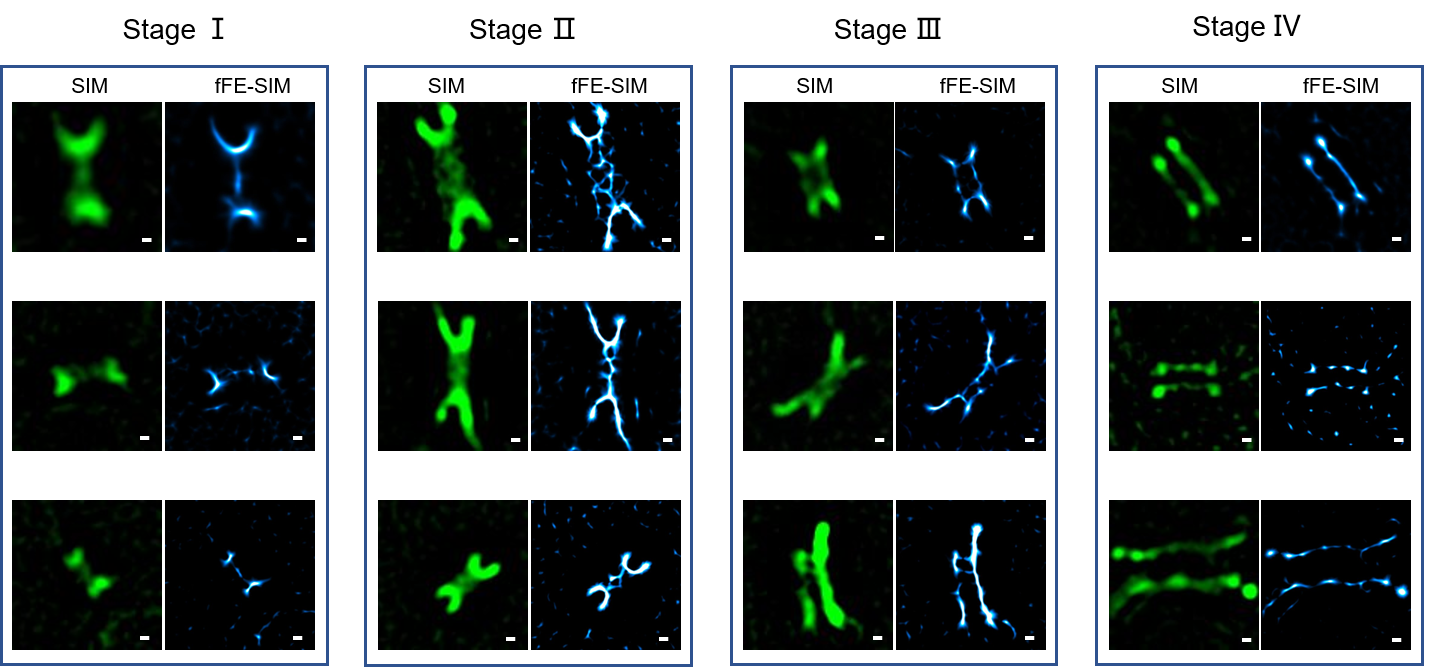

Supplement: Supplementary file 1 [file microorganisms-09-02255-s001.zip › si/S4.tif]
